# Unraveling Macrophage Polarization: Functions, Mechanisms, and “Double-Edged Sword” Roles in Host Antiviral Immune Responses

**DOI:** 10.3390/ijms252212078

**Published:** 2024-11-10

**Authors:** Meng Yao, Meilin Li, Dingkun Peng, Yijing Wang, Su Li, Ding Zhang, Bo Yang, Hua-Ji Qiu, Lian-Feng Li

**Affiliations:** 1 State Key Laboratory for Animal Disease Control and Prevention, Harbin Veterinary Research Institute, Chinese Academy of Agricultural Sciences, Harbin 150069, China; a13678602285@163.com (M.Y.); m18037901611@163.com (M.L.); pengdk11@163.com (D.P.); 15039269261@163.com (Y.W.); lisu@caas.cn (S.L.); 2 College of Veterinary Medicine, Shanxi Agricultural University, Taigu, Jinzhong 030801, China; zdlucky2014@sina.com (D.Z.); bo_yang@sxau.edu.cn (B.Y.)

**Keywords:** macrophages, macrophage polarization, viruses, antiviral immunity, immune escape

## Abstract

Numerous viruses that propagate through the respiratory tract may be initially engulfed by macrophages (M*φ*s) within the alveoli, where they complete their first replication cycle and subsequently infect the adjacent epithelial cells. This process can lead to significant pathological damage to tissues and organs, leading to various diseases. As essential components in host antiviral immune systems, M*φ*s can be polarized into pro-inflammatory M1 M*φ*s or anti-inflammatory M2 M*φ*s, a process involving multiple signaling pathways and molecular mechanisms that yield diverse phenotypic and functional features in response to various stimuli. In general, when infected by a virus, M1 macrophages secrete pro-inflammatory cytokines to play an antiviral role, while M2 macrophages play an anti-inflammatory role to promote the replication of the virus. However, recent studies have shown that some viruses may exhibit the opposite trend. Viruses have evolved various strategies to disrupt M*φ* polarization for efficient replication and transmission. Notably, various factors, such as mechanical softness, the altered pH value of the endolysosomal system, and the homeostasis between M1/M2 M*φ*s populations, contribute to crucial events in the viral replication cycle. Here, we summarize the regulation of M*φ* polarization, virus-induced alterations in M*φ* polarization, and the antiviral mechanisms associated with these changes. Collectively, this review provides insights into recent advances regarding M*φ* polarization in host antiviral immune responses, which will contribute to the development of precise prevention strategies as well as management approaches to disease incidence and transmission.

## 1. Introduction

Macrophages (M*φ*s) are multifunctional immune cells in the immune system, characterized by their long lifespan and phagocytic activities [1]. They play crucial roles in the host’s antiviral immune response and perform various functions within the body, including communication and defense, and they serve as part of early warning mechanisms. Tracking their early development poses significant challenges. However, advances in single-cell omics techniques have shed light on the origin of human M*φ*s. These studies have identified early yolk sac-derived myeloid-biased progenitors (YSMPs) produced from non-hematopoietic stem cells as the source of M*φ*s [2]. While it is traditionally believed that M*φ*s originate solely from bone marrow monocytes, recent research has revealed two distinct sources: monocyte-derived M*φ*s (MDMs) and tissue-resident macrophages (TRMs) [3]. TRMs are independent of the hematopoietic system, have the ability to self-renew, and colonize tissues during early embryonic development.

M*φ*s exert antiviral functions in vivo, while they can undergo polarization into two major activation phenotypes mediated by the cytokines produced by helper T cells in vitro. The diverse activated phenotypes of M*φ*s are determined by their heterogeneous origins and polarization states [4]. The plasticity of M*φ*s determines their ability to polarize into different phenotypes under the effects of various stimuli. In an inflammatory environment, M*φ*s can be polarized toward the classically activated M1 phenotype (pro-inflammatory) or the alternatively activated M2 phenotype (anti-inflammatory) [5].

Viral infections are closely associated with M*φ*s. On one hand, M*φ*s are among the primary targets of numerous pathogenic microorganisms, serving as infection sites for a diverse range of viruses. For instance, African swine fever virus (ASFV) and porcine reproductive and respiratory syndrome virus (PRRSV) exhibit a preference for infecting cells within the M*φ*s lineage and specifically target distinct subpopulations of M*φ*s located in the placenta, lymphatic organs, and lungs [6]. On the other hand, as crucial components of both innate and adaptive immunity, M*φ*s play a pivotal role in producing various cytokines that combat or facilitate the invasion of pathogenic microorganisms and modulate inflammation.

## 2. The Characteristics of M*φ*s

M*φ*s, a classical type of innate immune cells, are widely distributed throughout the body and play diverse roles, including those of ‘communicator’, ‘death squad’, and ‘sentinel’. However, the investigation of these cells poses considerable challenges owing to their limited availability during early development and the difficulties in tracing them.

### 2.1. The Origin and Classification of Mφs

**Figure 1:**
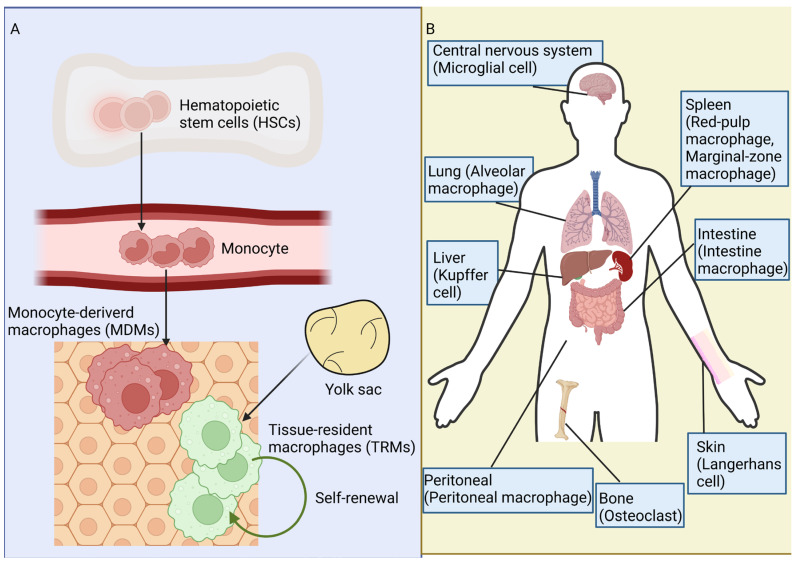
Origin and distribution of macrophages (M*φ*s). (**A**) The origin of M*φs*. Hematopoietic stem cells (HSCs) in the bone marrow can differentiate into monocytes, which subsequently enter the blood and mature. These circulating monocytes are capable of migrating through blood vessels into tissue, where they differentiate into monocyte-derived Mφs (MDMs). Tissue-resident macrophages (TRMs) are mainly generated by the self-renewal and regeneration of existing M*φs*. Many TRMs are established during embryonic development rather than adult blood monocytes, and they operate independently of one another. (**B**) The distribution of M*φs* in different organs. Upon migrating to specific tissues, M*φs* differentiate into tissue-specific M*φs*. These include microglia in the central nervous system, Kupffer cells in the liver, Langerhans cells in the epidermis, osteoclasts within the skeletal system, alveolar M*φs* in the lung, histocytes in connective tissues, and metallophilic cells in the spleen.

M*φ*s originate from two primary sources, MDMs and TRMs (Figure 1A). MDMs differentiate from circulating monocytes and usually migrate to specific tissues in response to inflammation or tissue damage. In contrast, TRMs are localized in specific tissues, exhibiting self-renewing capabilities independent of the hematopoietic system [7]. TRMs fulfill specific physiological functions and are ubiquitously distributed across various tissues (Figure 1B). They establish close and stable associations with specific tissue cells [5], such as Langerhans cells in the epidermis, Kupffer cells in the liver, and microglia in the central nervous system [8]. While M*φ*s originate from bone marrow hematopoietic monocytes during adulthood and can be found in the small intestine and skin, the majority of TRMs is not derived from post-adult bone marrow monocytes but rather originate from the yolk sac or fetal liver during embryonic development, subsequently undergoing maturation. Recent years have witnessed fresh perspectives on the genesis of TRMs. The observation that the starfish lacks a vascular system yet possesses embryonic M*φ*s has prompted researchers to question the assumption that M*φ*s exclusively originate from blood monocytes, as well as the theoretical conclusion that inflammation cannot occur outside of blood vessels. Subsequent investigations have revealed that a significant proportion of TRMs arise during embryonic development rather than being derived solely from adult blood monocytes [9,10,11]. Consequently, TRMs are partially generated through both the self-renewal of resident M*φ*s and the recruitment of circulating monocytes. Multiple studies have demonstrated the self-renewal capacity of TRMs. For example, human Langerhans cells were found to proliferate even 4.5 years after transplantation [12], which is consistent with similar findings in mice models [13]. As ‘sentinels’, M*φ*s respond rapidly to stimuli through polarization and by contributing to the biological process. Resident M*φ*s play pivotal roles in organogenesis, promote tissue regeneration following damage, and contribute to tissue metabolism as well as defense against infectious disease [14]. Distinct populations of tissue M*φ*s exhibit different functions (Table 1).

### 2.2. The Classification of Mφ Polarization

**Figure 2:**
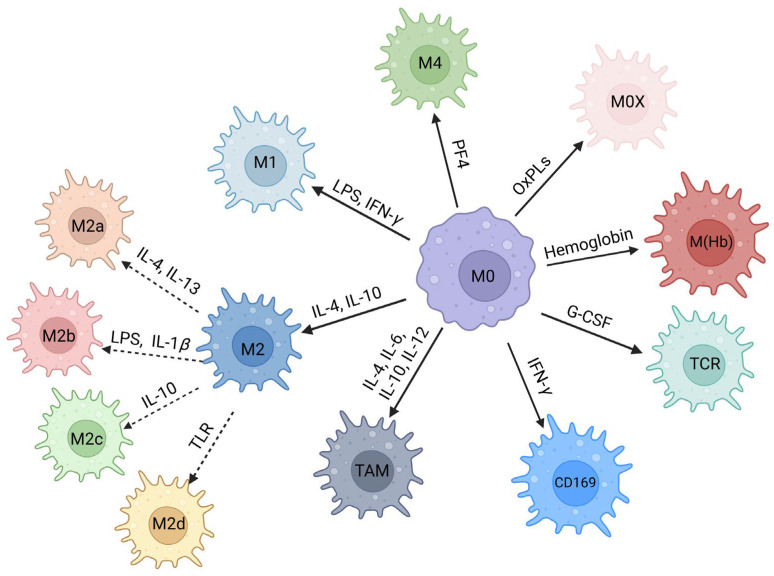
Classification of macrophage (M*φ*) polarization. Under stimulation by different factors, M0 M*φ*s can be polarized into different phenotypes. M1 M*φ*s are classically activated in response to lipopolysaccharides (LPSs) and interferon gamma (IFN-*γ*). Conversely, upon stimulation by interleukin 4 (IL-4) and IL-10, M2 M*φ*s are activated instead, which can be further divided into M2a, M2b, M2c, or M2d phenotypes depending on the specific stimuli. In the presence of IFN-*γ* and granulocyte colony-stimulating factor (G-CSF), M*φ*s may be polarized into CD169^+^ M*φ*s and TCR^+^ M*φ*s, respectively. Under certain conditions, such as within the tumor microenvironment, tumor-associated M*φ*s are polarized. In atherosclerosis, they are polarized into M4, Mox, and M(Hb) phenotypes by platelet factor 4 (PF4), oxidized phospholipids (OxPLs), and hemoglobin. TLR—toll-like receptor.

The effects of M*φ*s on various functional phenotypes, which are generated by unique microenvironments and signals under particular circumstances, are collectively known as M*φ* polarization [25]. M*φ*s polarize into different phenotypes in response to different factors, and phenotypic changes in cytokines were observed following stimulation in vitro [26] (Figure 2). The polarized macrophages can be broadly classified into two primary groups: classically activated M*φ*s (M1) and alternatively activated M*φ*s (M2) [27]. Although this dichotomy offers a useful framework for elucidating M*φ* heterogeneity, recent advancements in single-cell technologies and systems biology have uncovered a continuum of activation states that transcend the M1/M2 paradigm [28]. In addition to that, there are CD169^+^ M*φ*s, tumor-associated macrophages (TAMs), and TCR^+^ M*φ*s [29]. Although the properties of these three M*φ* subtypes remain unclear, evidence suggests that they play significant roles in certain human illnesses. Furthermore, atherosclerosis has been linked to multiple other M*φ* phenotypes, which are recognized through the stimulation of CXCL4 (M4), oxidized phospholipids (Mox), hemoglobin/haptoglobin complexes [HA-mac/M(Hb)], and heme (Mhem) [30]. This review primarily focuses on the physiological and clinical roles of the M1 and M2 phenotypes (Table 2).

#### 2.2.1. M1 Phenotype

It has been proposed that classical activation of the M1 phenotype is primarily induced by two kinds of stimuli [60]. The first category comprises small molecules, such as toll-like receptor (TLR) ligands, interferon gamma (IFN-*γ*), lipopolysaccharides (LPSs), and granulocyte-macrophage colony-stimulating factor (GM-CSF) [31]. The second category encompasses pathogen-associated molecular patterns (PAMPs), which drive M*φ* polarization toward the M1 phenotype. M1 M*φ*s exhibit a pro-inflammatory phenotype with the capability to eliminate pathogens and produce pro-inflammatory cytokines, such as tumor necrosis factor alpha (TNF-*α*), interleukin 1beta (IL-1*β*), IL-12, and IL-23 [32]. They also secrete increased levels of reactive oxygen species (ROS) and possess stronger antigen-presenting ability [33]. The key surface markers of M1 M*φ*s mainly include inducible nitric oxide synthase (iNOS), CD68, CD80, CD86, and Fc receptors (CD32A, CD32B, CD64, and CD16) [34,35,36,37,38].

#### 2.2.2. M2 Phenotype

M2 M*φ*s are activated through an alternative pathway that is distinct from the classical activation pathway [29]. These M2 M*φ*s can be polarized by various stimulating factors, including IL-4, IL-10, and IL-13, as well as fungi and worms [43]. Furthermore, M2 M*φ*s are subdivided into four subgroups: M2a, M2b, M2c, and M2d [44]. Each phenotype exhibits unique cell surface markers, secreted cytokines, and biological functions. Specifically, M2a M*φ*s are activated by IL-4 or IL-13 and possess anti-inflammatory and reparative effects that promote cell proliferation and repair tissue damage. In contrast, M2b M*φ*s are activated by immune complexes, including TLR ligands and IL-1*β*, and secrete both pro-inflammatory and anti-inflammatory cytokines, such as TNF-*α*, IL-1*β*, IL-6, and IL-10, regulating T-cell immune responses and promoting inflammatory responses [50]. Meanwhile, M2c M*φ*s, also known as inactivated M*φ*s, are induced by glucocorticoids, IL-10, and transforming growth factor beta (TGF-*β*). These cells assist in the phagocytosis of apoptotic cells by secreting IL-10, TGF-*β*, C-C motif ligand 16 (CCL16), and CCL18 [51]. Lastly, M2d M*φ*s, which are induced by TLR antagonists, cause the secretion of vascular endothelial growth factor (VEGF) and IL-10, which in turn promote angiogenesis and the growth of tumors [52]. Notably, compared to other phenotypes within this classification system, M2 M*φ*s exhibit higher phagocytic activity and elevated expression levels of scavenger, mannose, and galactose receptors [61]. The main surface markers of M2 M*φ*s include arginase 1 (Arg-1), scavenger receptors such as CD163 and macrophage scavenger receptor 1 (MSR1), mannose receptor (CD206), CD9, major histocompatibility complex class II (MHC-II), triggering receptor expressed on myeloid cells 2 (TREM2), etc. [45,46,47,48].

#### 2.2.3. Other Phenotypes

Whether or not TAMs are a subgroup of M*φ*s remains highly debated, primarily because these cells do not exist in a homeostatic environment but rather survive in the tumor microenvironment (TME). TAMs are composed of both TRMs and infiltrating MDMs [53]. Accordingly, it is unsurprising that TAMs are extremely heterogeneous, with at least five to seven identifiable subgroups. Depending on the stimulus and microenvironment, TAMs can be phenotypically differentiated into either M1 or M2 M*φ*s driven by tumor-derived environmental factors, such as IL-10 and IL-4. Furthermore, TAMs secrete various enzymes, which contribute to the invasion and metastasis of tumor cells [54].

CD169^+^ M*φ*s that infiltrate brain tumors originate from blood monocytes, lymph nodes, the liver, and the spleen; these cells possess the capacity to enhance anti-tumor immune responses through their interaction with red blood cells [55]. The T-cell receptor (TCR), a broadly antigen-specific molecule that forms a complex with CD3, is essential for antigen recognition. Recent publications have documented the presence of TCR^+^ M*φ*s in both humans and mice. Notably, Fuchs et al. reported the accumulation of TCR^+^ M*φ*s in atherosclerotic lesions observed in both species [56]. However, little is currently known about this specific subpopulation of TCR^+^ M*φ*s. Research indicates that the polarization of M*φ*s toward the M4 phenotype can be induced by platelet factor 4 (PF4) [57]. Additionally, exposure to oxidized phospholipids leads to polarization into a novel phenotype called ‘Mox’, which markedly differs from both the M1 and M2 phenotypes, exhibiting distinct gene expression patterns [58]. Simultaneously, under stimulation by hemoglobin (Hb), M*φ*s will polarize into an alternative state known as M(Hb) [59].

### 2.3. Functions of Polarized Mφs

Under stress conditions, polarized M*φ*s intricately regulate and respond to diverse stimuli, thus playing pivotal roles in the pathogenesis of inflammation and diseases as well as in the extent of tissue and organ repair. M1 M*φ*s are primarily involved in inflammatory responses, microbicidal functions, and tumoricidal activities. In contrast, M2 M*φ*s are predominantly responsible for modulating inflammatory responses, adaptive immunity, tissue remodeling and repair processes, debris clearance, and tumor progression.

#### 2.3.1. Inflammatory Responses and Tissue Repair

When tissues are infected or mechanically damaged, invading microorganisms and dead cells will activate damage-associated molecular patterns (DAMPs) and PAMPs, causing inflammation [62]. Among these, M1 M*φ*s are responsible for the phagocytosis and elimination of pathogens, as well as for producing cytokines like IL-1*β* and TNF-*α*, which are important in protecting the body against pathogens and cell damage, and in reducing the effects of harmful substances. In later stages, activated by IL-4 and IL-13, M*φ*s at the injury site exhibit an M2 phenotype [63]. M2 M*φ*s possess various biological functions, including promoting immune cell activity and apoptosis as well as accelerating the repair of damaged tissues, etc.; these functions are vital for immune regulation and tissue repair within the body. In the process of inflammation, the coordination and interactions between M1 M*φ*s and M2 M*φ*s play important roles in immune responses. These patterns recruit and activate a variety of immune cells including neutrophils, M*φ*s, natural killer (NK) cells, B cells, and T cells to orchestrate comprehensive immune responses [64]. M*φ*s and neutrophils serve as the initial responders to infections [65]. While multiple cell types participate in tissue healing processes, due to their highly adaptable programming capabilities, M*φ*s have been shown to play crucial regulatory roles throughout all stages of repair and fibrosis [66,67].

#### 2.3.2. Developmental Regulation of Tumors and Cancers

M*φ*s are involved in shaping the TME [7]. TAMs refer to M*φ*s that are recruited into TMEs, which have fundamental impacts on the occurrence and development of tumors. M*φ*s are plastic and, once absorbed by malignant tumors, they coordinate multiple interactions in the TME, especially playing key roles in matrix remodeling, angiogenesis, metastasis, and tumor development, thus driving the evolution of the cancer ecosystem [68]. It has been shown that the poor prognosis of solid tumors, including in breast cancer [69], bladder cancer [70], head and neck cancer [71], glioma [72], melanoma [73], and prostate cancer [74], is associated with M*φ*s infiltration. However, in colorectal and gastric cancers, high levels of M*φ*s infiltration are associated with a better prognosis [75]. These apparently opposite effects may be related to M*φ* plasticity and the phenotypic and functional heterogeneity of various cancers. Therefore, several therapeutic targets of M*φ*s in anti-cancer therapy have been identified, including TAM depletion, the inhibition of new TAM differentiation, or the reinduction of TAMs to activate cancer cell phagocytosis.

#### 2.3.3. Immune Responses to Pathogenic Microorganisms

**Figure 3:**
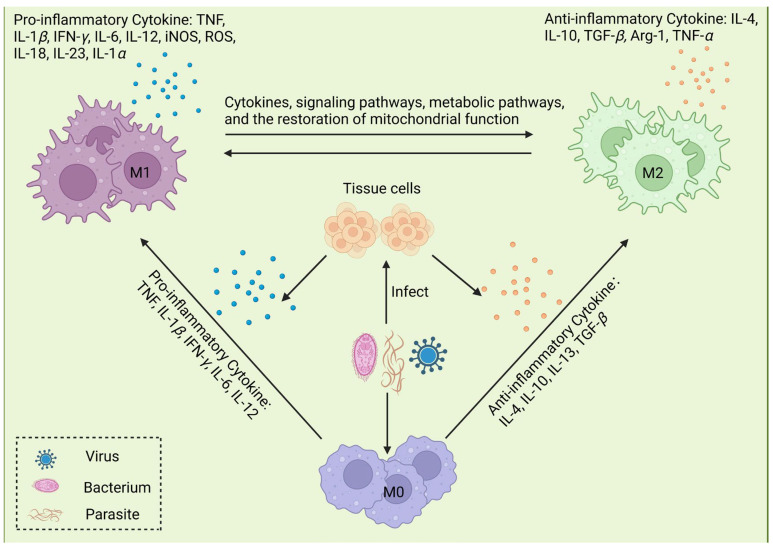
Mechanisms of microorganism-induced macrophage (M*φ*) polarization. Following invasion of the body by pathogenic microorganisms (bacteria, viruses, parasites, etc.), these pathogens infect corresponding tissue cells, which subsequently secrete a series of cytokines, such as tumor necrosis factor-alpha (TNF-*α*), TNF-*β*, interleukin-6 (IL-6), IL-12, IL-1*β*, IL-4, and IL-10. With the secretion of cytokines, M*φ*s are recruited to the injury site, where they are polarized into different phenotypes under stimulation by different cytokines, thus participating in the immune response and maintaining body homeostasis. M1 M*φ*s secrete pro-inflammatory cytokines, including TNF, IL-1*β*, interferon gamma (IFN-*γ*), IL-6, IL-12, inducible nitric oxide synthase (iNOS), and reactive oxygen species (ROS), which participate in the early inflammatory response, while M2 M*φ*s can release IL-10, TGF-*β*, and other anti-inflammatory cytokines.

M*φ*s play important roles in infections by pathogenic microorganisms (Figure 3). M*φ*s identify and present exogenous antigens in MHC-I and MHC-II to T cells, which recognize the MHC–antigen complex via their T-cell receptor [76]. M1 M*φ*s possess robust antigen-presenting activity and express elevated levels of MHC-II, which significantly enhances their capacity to directly present antigens to T cells, inclining M*φ*s toward promoting inflammation and immune activation [77]. In contrast, M2 M*φ*s regulate the immune response by secreting anti-inflammatory cytokines and expressing specific surface receptors, allowing them to suppress inflammation and facilitate tissue repair [78]. Upon infection with *Salmonella typhi* and *Listeria monocytogenes*, M*φ*s are polarized into M1 M*φ*s, releasing a large number of inflammatory factors to eliminate the invading pathogens and induce adaptive immunity [79]. However, excessive inflammatory factors can cause an ‘inflammatory cytokine storm’, which leads to sepsis [80,81]. To mitigate excessive inflammatory responses, M*φ*s utilize inhibitory signaling molecules and then are activated into M2 M*φ*s to promote the regression of inflammation and the repair of tissue damage [82]. It has been established that during the recovery phase of typhoid patients, there is a progressive transition in M*φ* gene expression from the M1 to M2 phenotype until inflammation and tissue damage are resolved, ultimately resulting in the return of M2 M*φ*s to their quiescent state [83]. In response to parasitic infections, M*φ*s exhibit dynamic polarization states characterized first by polarization toward M1 M*φ*s and then by transformation into M2 M*φ*s. M2 M*φ*s can inhibit T-cell responses, regulate fibrosis, and form multinucleated giant cells in granulomatous lesions. Notably, as a marker molecule of M2 M*φ*s, Arg-1 plays a significant role in anti-infection processes for some parasites [84]. For example, following *Schistosoma mansoni* infection, Arg-1 effectively regulates Th2-mediated fibrosis, Th1/Th17-mediated intestinal injury, iNOS production, and endotoxemia [85].

M*φ*s possess a unique dual identity and therefore play irreplaceable roles during viral infection. Previous studies have primarily focused on the phagocytosis and antigen presentation capabilities of M*φ*s. However, recent studies have indicated that M*φ*s with different activation modes have a dual regulatory effect on the inflammatory response of the body after viral infection. The polarization state of M*φ*s is closely related to both the occurrence and outcome of viral infectious diseases. Therefore, we focus on exploring the mechanisms underlying M*φ* polarization during viral infections.

## 3. Regulatory Mechanisms of M*φ* Polarization

M*φ* polarization is a dynamic process regulated by various factors, including transcription factors, signaling pathways, and metabolic reprogramming [86]. Transcription factors modulate the transcription rate to adjust the number of gene products; however, these transcription factors themselves have also been adjusted [87].

### 3.1. Transcription Factors and Signaling Cascades

**Figure 4:**
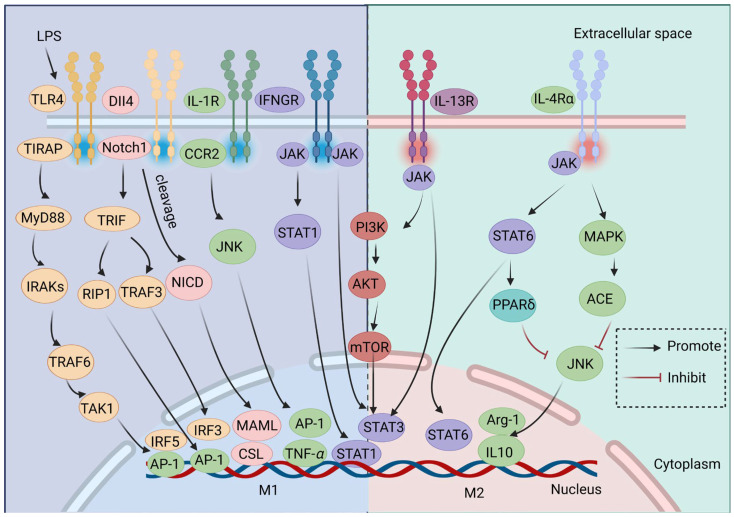
Signaling pathways regulating macrophage (M*φ*) polarization. Distinct transcription factors and signaling pathways influence the polarization of M1 and M2 M*φ*s. The JAK/STAT, PI3K/Akt, JNK, Notch and TLR signaling pathways serve as primary examples of these regulatory mechanisms. Interferon gamma (IFN-*γ*) binds to its receptor, interferon gamma receptor (IFNGR), leading to the polarization of M*φ*s toward the M1 phenotype by phosphorylating STAT1 and activating the JAK/STAT1 signaling pathway. This process also initiates the transcription of IFN-stimulated genes. The binding of interleukin 4 (IL-4) or IL-13 to their respective receptors results in the activation of STAT3 or STAT6, respectively; this promotes the transcription of anti-inflammatory cytokines that drive M*φ*s toward an anti-inflammatory M2 phenotype. Additionally, upon interaction with its receptor, the Notch protein is activated and the Notch intracellular domain (NICD) is translocated to the nucleus. This event subsequently enhances the production of pro-inflammatory cytokines and M1-related encoding genes like mastermind-like transcriptional coactivator (MAML) and CBF1/Su(H)/LAG-1 (CSL). There are no precise limits to the PI3K/Akt/mTOR signaling pathway’s ability to control M1 and M2 M*φ*s. IL-1 and IL-4 activate the JNK signaling pathway, which causes M*φ* polarization toward the M1 and M2 phenotypes, respectively. The MyD88-independent pathway of the TLR4 signaling pathway mainly activates interferon regulatory factor 3 (IRF3) to induce type I IFNs. DLL4, delta-like ligand 4; CRR2, C-C motif chemokine receptor 2; JNK, c-Jun N-terminal kinase; JAK, Janus kinase; STAT, signal transducer and activator of transcription; MAPK, mitogen-activated protein kinase; mTOR, mammalian target of rapamycin; Arg-1, arginase 1; AP-1, activator protein 1; ACE, angiotensin-converting enzyme.

The regulation of transcription factors and signaling pathways is mutually dependent. M*φ* polarization represents a complex multifactorial interaction process, governed by an array of signaling cascades (Figure 4). Members of the signal transducer and activator of transcription (STAT) protein family are key transcription factors that mediate M*φ* M1/M2 polarization. STAT1 serves as an important mediator of IFN-*γ*-induced M1 M*φ* polarization, with its activation promoting inflammatory responses in various diseases. Conversely, STAT3 activation regulates M*φ* polarization within the tumor microenvironment. Additionally, STAT6 is a key transcription factor for IL-4- or IL-13-mediated M2 M*φ* polarization, activating the transcription of genes typical of this phenotype [88].

It has been demonstrated that IFN-*γ* stimulates the JAK/STAT1 pathway, causing the polarization of M*φ*s toward the M1 phenotype [39]. Nevertheless, both IFN-*α* and IFN-*β* can inhibit STAT1 phosphorylation and subsequent M1 M*φ* polarization through negative feedback regulation [40]. Furthermore, stimulation of the IL-10/JAK/STAT3 signaling pathway may reduce or eliminate inflammation by promoting the polarization of M2 M*φ*s, thereby promoting regenerative tissue repair [49].

The phosphatidylinositol-3 kinase (PI3K) and protein kinase B (Akt) signaling pathways have been reported to regulate segmental M*φ* polarization. One study demonstrated that methylene THF dehydrogenase 2 (MTHFD2), a carbon-metabolizing enzyme, interacts with phosphatase and tensin homolog (PTEN) to inhibit PTEN’s PIP3 phosphatase activity while enhancing downstream Akt activation, thereby inhibiting the polarization of IFN-*γ*-activated M1 M*φ*s but enhancing the polarization of IL-4-activated M2 M*φ*s both in vivo and in vitro [41].

The regulation of cellular development, inflammatory responses, and cardiovascular disorders is attributed to the superfamily of mitogen-activated protein kinases (MAPKs), which includes the c-Jun N-terminal kinase 2 (JNK2) signaling pathway. The JNK signaling pathway has been demonstrated to play dual roles in regulating M*φ* polarization, with one aspect serving as its primary function. Activation of JNK2 signaling in M*φ*s is required for the pro-inflammatory phenotype induced by vitamin D deficiency [89]. Additionally, M*φ* MSR1 activates the JNK signaling pathway through K63 ubiquitination in IL-4-activated M*φ*s, thereby promoting the transformation of M*φ*s from an anti-inflammatory state to a pro-inflammatory state, and the opposite is observed in the absence of MSR1 [90].

The Notch signaling pathway exerts a crucial role in regulating the polarization of M*φ*s [91]. Activation of Notch signaling drives M*φ* polarization toward the M1 phenotype, thereby promoting inflammation, while inhibition of this signaling pathway induces M*φ* polarization toward the M2 phenotype and suppresses inflammatory responses.

During M*φ* polarization, LPSs activate two signaling pathways via TLR4: the MyD88-dependent and -independent pathways. The MyD88-dependent pathway primarily activates nuclear factor kappa B (NF-*κ*B), which promotes the expression of inflammatory cytokines, such as IL-1*β*, IL-6, and TNF-*α*. Conversely, the MyD88-independent pathway mainly activates interferon regulatory factor 3 (IRF3) to induce type I IFNs [92]. The Notch1 signaling pathway can modulate TLR4 signaling independently of MyD88. It has been demonstrated that the LPSs-induced activation of TLR4 through the Notch1 pathway facilitates the transition of M*φ*s into the M1 phenotype and promotes cytokine production [42].

### 3.2. Metabolic Reprogramming

A growing number of findings highlight the critical role of metabolic reprogramming in M*φ* activation, whereby metabolic pathways not only provide energy but also regulate the phenotypes and functions of M*φ*s. The first characteristic that is used to identify the M*φ* subpopulation is changes in amino acid metabolism. M1 M*φ*s utilize iNOS to convert arginine to nitric oxide (NO), whereas in IL-4-triggered M2 M*φ*s, arginine is processed by arginase-1. Significantly, distinct metabolic pathways are required to meet the energy needs of both M1 and M2 M*φ*s. The pentose phosphate route and the aerobic glycolysis pathway are necessary for the regulation of polarization because they supply necessary energy [93]. Since a large number of intermediate metabolites, mainly lactic acid, can be produced during the glycolysis process, the importance of lactic acid is obvious [94]. Studies have shown that lactic acid serves as a substrate to modify the lysine site of histone H3 (H3K18) in M*φ*s and regulate the polarization of M*φ*s toward the M1 phenotype in the late stage of infection [95]. Furthermore, after LPSs-induced M*φ* polarization to the M1 phenotype, histone lactate modification inhibits M*φ* polarization toward this state [96]. Infiltrating macrophages create an inflammatory environment by releasing superoxide derived from nicotinamide adenine dinucleotide phosphate (NADPH) oxidase and pro-inflammatory cytokines [97]. NADPH oxidase 4 (NOX4) promotes glycolysis in microglial cells through ROS, thereby accelerating M1 polarization and the expression of inflammatory factors [98]. This suggests a link between M*φ* metabolism and inflammatory phenotypes along with additional regulatory mechanisms governing M*φ* polarization through metabolic pathways.

## 4. Viral Infection-Induced Polarization of M*φ*s

### 4.1. Influence Factors

The phenotype of M*φ*s is not static but rather dynamic, with their polarization significantly influenced by both the tissue microenvironment and disease states [99]. M1 and M2 M*φ*s possess the ability to adapt their phenotypes in response to specific tissue microenvironments. In summary, during the acute phase of viral infection, M*φ*s are polarized toward the M1 phenotype, which can promote inflammation and assist the body in clearing pathogens. However, their overactivation can cause an inflammatory cytokine storm and aggravate the immunopathological damage of tissues. Furthermore, the polarization ratio of M1 M*φ*s is positively correlated with the severity of disease. As viral infection progresses, M*φ*s become polarized toward the M2 phenotype, playing an immune-regulatory role by secreting various anti-inflammatory factors and participating in tissue repair processes [100,101]. M*φ* polarization is not based only on the two extremes of M1/M2 M*φ*s, but rather on a continuous M*φ* activation spectrum, which entails a dynamic development process. The transition from the pro-inflammatory state of M1 M*φ*s to the regulatory or anti-inflammatory state of M2 M*φ*s is thought to help improve functional outcomes and restore homeostasis [102]. Therefore, following viral clearance, it is crucial for the immune cells to be cleared in time [103,104]. If M*φ*s are depleted at an early stage after injury occurs, inflammatory responses will be greatly weakened [105]. Meanwhile, inhibiting the directional polarization of M1 M*φ*s is beneficial to the control of the inflammatory response [106].

#### 4.1.1. Virulence Factors

To some extent, phenotypic tropism is influenced by the virulence of the virus strain, which is mainly reflected in the fact that pathogenic virus strains inhibit the antiviral response of M1 M*φ*s and tilt M*φ*s toward the M2 phenotype, while attenuated virus strains induce polarization toward the M2 phenotype. Previous studies have demonstrated that Junin virus (JUNV) elicits the expression of distinct receptor molecules and selectively regulates cytokine production, with one strain showing elevated levels of TNF-*α*, IL-10, and IL-12, while the other strain exclusively induces higher levels of IL-6. Infection with these two strains resulted in the polarization of M*φ*s toward the M1 and M2 phenotypes, respectively [107]. After infection with a virulent Newcastle disease strain (ND), M*φ*s underwent polarization into a mixed M1/M2 phenotype, facilitating rapid viral replication within these cells. In contrast, although the attenuated ND strain exhibited early-stage proliferation similar to the virulent strain, its later-stage replication was significantly hindered due to its inability to hijack M*φ*s and induce substantial M1 or M2 polarization based on the specific requirements [108]. There is evidence that the sensitivity of M*φ*s to different subtypes of influenza virus (IV) varies, and most highly pathogenic H5N1 subtype avian influenza virus (HPAIV) isolates can effectively induce M1 polarization of M*φ*s [109].

#### 4.1.2. Viral Components

Viral components, such as viral particle surface proteins, bind to host cell receptors and can influence the polarization of M*φ*s. For example, mutations in the receptor-binding proteins of the spike (S) protein of SARS-CoV-2 can enhance the virus’s affinity to human receptors and thus affect the function of host cells. Notably, mutations in the receptor-binding domain (RBD) of SARS-CoV-2, such as D614G, have been found to enhance the structural stability of the S protein, to show higher human ACE2 affinity, and have higher infectiveness [110]. Additionally, some viral proteins can act directly on M*φ*s to induce their polarization. For example, the NS1 protein of IVs can inhibit the IFN signaling pathway and promote M2 M*φ* polarization [111].

### 4.2. Polarization to M1 Phenotype

ROS are essential for the polarization of M1 M*φ*s. Numerous studies have demonstrated that ROS can activate transcription factors that promote inflammatory responses, like NF-*κ*B and AP-1 [112]. Furthermore, the activation of NF-*κ*B and p38 MAPK signaling pathways upregulates the expression of pro-inflammatory chemokines/cytokines and adhesion molecules in M*φ*s, thereby promoting the polarization of M*φ*s to the M1 phenotype [113,114]. Advanced glycation end products (AGEs) heighten the cardiovascular risk in individuals with diabetes by stimulating inflammation and facilitating the formation of atherosclerosis [115]. AGEs can enhance M*φ* polarization into the M1 M*φ*s via the activation of the RAGE/NF-*κ*B pathway [116]. During the process of M*φ* polarization, the secretion of proteins in the complement and coagulation pathways assumes a crucial role in the activation and polarization of M*φ*s, and they are involved in the regulation of M*φ* polarization by modulating the inflammatory response and the recruitment of immune cells. Complement components C3a, C5a, and C5b-9 regulate cytokine production in M*φ*s via diverse signaling pathways, tending toward pro-inflammatory M1 M*φ*s [117]. The complement component C1q or C3b governs the production of anti-inflammatory M2 M*φ*s and blocks pro-inflammatory signals [118]. There exists a connection between the clotting system and the inflammation regulated by cells of the innate immune system. Thrombin induces M*φ*s to polarize toward M1 M*φ*s characterized by the expression of pro-inflammatory cytokines and chemokines. This effect is at least partly mediated by protease-activating receptor 1 (PAR-1) [119].

Viral RNA or DNA components serve as primary PAMPs, which are readily capable of inducing an M1 phenotype in M*φ*s [120,121]. To some extent, viral infection induces M*φ*s to be polarized toward the M1 phenotype due to the secretion of pro-inflammatory cytokines, thus promoting the spread of the virus, which is a vicious cycle [122]. Upon infection by a pathogen, M*φ*s communicate with each other by secreting cytokines that cause more M*φ*s to polarize and reach the site of infection to clear the infection. After entering host target cells through receptor-mediated endocytosis and proliferation, viruses release more virions by means of budding or induced programmed cell death. Once the released viruses are recognized by the pattern recognition receptor on immune cells, through a series of signal transduction processes, a large number of cytokines are released to activate more M*φ*s to participate in viral elimination.

Numerous studies have demonstrated that M1 M*φ*s serve as the first line of defense against infections, while some specific viruses are able to evade the immune system by preventing M*φ* polarization into the M1 phenotype. This inhibition of M1 M*φ* polarization facilitates the immune evasion of pathogenic viruses, subsequently promoting their proliferation and dissemination within the host. However, the precise mechanisms of inhibiting the polarization of M*φ*s to the M1 phenotype to achieve immune escape remain unclear. In summary, the role of M1 M*φ*s in viral replication is complex, involving the synergistic action of multiple mechanisms.

### 4.3. Polarization to M2 Phenotype

Viruses have evolved multiple mechanisms to induce M2 M*φ* polarization. Firstly, they promote M2 M*φ* polarization by upregulating the expression of inhibitory receptors, including programmed cell death 1 (PD-1), PD-1 ligand (PD-1L), and viral homologs of CD200 and CD47 [123]. Multiple studies have demonstrated that during infection with various viruses, such as chronic hepatitis C virus (HCV) and herpesvirus simplex 1 (HSV-1), the expression levels of PD-1 and PD-1L are upregulated, while the activation of IL-12 and STAT1 is inhibited, indicating the promotion of M2 M*φ* polarization [124]. At the same time, the homolog of CD200 inhibits M1 M*φ* polarization in an NF-*κ*B-dependent manner [125]. Simultaneous knockout of the viral homolog of CD47 helps M*φ*s migrate to the M1 phenotype and enhances antiviral response. Additionally, viruses can also induce M2 M*φ*s polarization by interfering with signaling pathways associated with this process. It is well established that STAT3 and its induction of downstream IL-10 are essential for promoting M*φ* polarization toward the M2 phenotype, while STAT1 is necessary for M1 M*φ* polarization [126]. The HCV E2 protein promotes IL-10 transcription and encourages M2 M*φ* polarization by inhibiting STAT1 activation and increasing STAT3 phosphorylation [127]. In addition, some viruses, including poxviruses and herpesviruses, enhance viral infection by directly inducing M*φ* polarization into the M2 phenotype [128]. Notably, NO’s antiviral response to M1 M*φ*s is crucial. Therefore, inhibiting its ability to produce NO is a key way of making M*φ*s polarize into the M2 phenotype. Research has shown that respiratory syncytial virus (RSV) infection raises NO levels, thereby preventing viral replication [129]. Simultaneously, many viruses can also induce M2 M*φ* polarization by reducing the production of pro-inflammatory cytokines.

## 5. Key Factors Affecting the Antiviral Response and Immune Escape of Polarized M*φ*s

**Figure 5:**
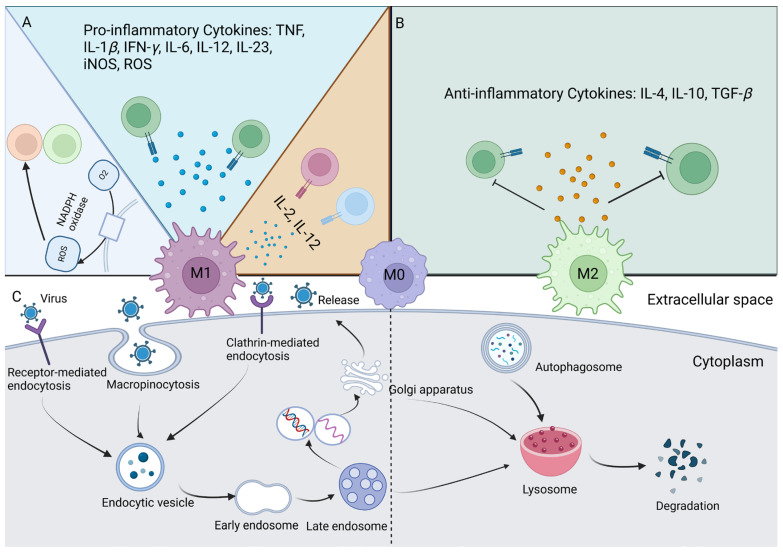
Dual identity of macrophage (M*φ*) polarization in antiviral immunity. (**A**) Antiviral mechanisms of M1 M*φ*s. During the process of viral infections, ROS produced by M1 M*φ*s can create a superoxide environment that stimulates the immune system and plays positively regulatory roles in virus clearance. M1 M*φ*s possess the ability to secrete pro-inflammatory cytokines, including IL-1-*β*, TNF-*β*, IFN-*γ*, IL-6, IL-12, IL-23, iNOS, ROS, etc. To further block the invasion of the virus, M1 M*φ*s can also activate additional immune cells to fight against the virus. (**B**) Immunosuppressive effects of M2 M*φ*s. M2 M*φ*s secrete anti-inflammatory cytokines, like TGF-*β* and IL-10. M2 M*φ*s, by controlling the activation of T cells, can induce immunosuppression and promote tumorigenesis while inhibiting the production of anti-tumor immune responses by T cells. (**C**) The endosomal–lysosomal system in M1 and M2 M*φ*s. Viruses primarily enter M*φ*s through endocytosis, including macropinocytosis, receptor-mediated endocytosis, and clathrin-mediated endocytosis (CME). Upon entry into the cell, the virus undergoes endocytosis, progressing through early and late endosomes before being degraded within the lysosomes. The acidic environment of endosomes and alkaline nature of lysosomes in M1 M*φ*s facilitate viral replication, leading to eventual viral escape. Conversely, the acidic lysosomes of M2 M*φ*s possess the ability to degrade the virus, thereby preventing its spread. ROS, reactive oxygen species; iNOS, inducible nitric oxide synthase.

The polarization of M*φ*s plays multifaceted and pivotal roles in antiviral immunity. On one hand, M*φ* polarization orchestrates a cascade of antiviral functions. M1 M*φ*s employ multiple strategies to combat viral invasion and have been demonstrated to play crucial roles in antiviral immunity against specific viral infections, including human immunodeficiency virus (HIV) and simian immunodeficiency virus (SIV) [130]. On the other hand, viruses employ diverse strategies to evade host immune responses by modulating M*φ* polarization states, thereby impeding or exploiting their antiviral capabilities for immune escape [131]. The effects of different virus strains on M*φ* polarization are multifaceted and may be influenced by viral virulence, infection stage, and host immune status. Nonetheless, M*φ*s exhibit a dual identity as immune cells with intricate intracellular antiviral mechanisms that necessitate further investigation (Figure 5). Therefore, maintaining a proper balance in the antiviral immune response is essential for ensuring effective antiviral response and protecting the health of the body [132].

### 5.1. Production of Reactive Species

Reactive species (RS), such as ROS and reactive nitrogen species (RNS), are produced by M1 M*φ*s in a strongly oxidizing environment [133]. ROS have long been understood to be a crucial modulator of M*φ* formation in the antiviral process. Superoxide anion (O^2−^), which promotes viral destruction, is produced during phagocytosis when the phagolysosomal membrane pumps electrons into the phagolysosome cavity [134]. Additionally, it has been documented that mitochondria can generate superoxides, which are then transferred to phagosomes, thereby enhancing phagocytic activity and facilitating the engulfment of invading viruses [135,136]. NO, the most critical RNS in M*φ*s, is synthesized when inducible nitric oxide synthase 2 (iNOS2) catalyzes the conversion of L-arginine [137]. However, NO generation varies depending on the type of M*φ*s and the expression level of iNOS2 [138]. In M*φ*s, pro-inflammatory cytokines including TNF-*α*, IFN-*γ*, or elements of the microbial cell wall trigger iNOS expression [139]. NO exhibits wide-range potent antiviral activities through various mechanisms. Activated M*φ*s were cytotoxic to tumor cells when L-arginine was introduced [140]. Subsequent findings verified that this killing mechanism required NO assistance provided from iNOS [141]. In M*φ*s, NO-mediated tumor rejection is induced by M1 M*φ* polarization, indicating their capacity to destroy cancer cells *via* NO production [142]. Moreover, NO indirectly modulates gene transcription through the regulation of various signaling pathways, including the phosphoinositide PI3K pathway [143]. The concentration and duration of NO transmission along with the redox species linked to NO all influence its effects on gene regulation. However, excessive production of these oxidative stress chemicals may harm host cells and interfere with regular physiological processes [144].

### 5.2. Secretion of Cytokines

M1 M*φ*s possess the ability to generate a significant amount of pro-inflammatory cytokines, including TNF-*α*, IL-1, IL-6, IL-8, and IL-12 [145]. These cytokines are engaged in the early inflammatory response, trigger antiviral immune responses, and exhibit antiviral action directly or indirectly. The recognition of tobacco mosaic virus (TMV) by M*φ*s occurs via TLR4, subsequently activating the MAPK and NF-*κ*B signaling pathways. This activation leads to the polarization of M*φ*s into the M1 phenotype, resulting in the production of pro-inflammatory cytokines and initiation of an immune response [146]. Several pro-inflammatory cytokines (IL-6, IL-1*β*, and TNF-*α*), IFN-*α*, and IFN-*γ* have been shown to directly inhibit hepatitis B virus (HBV) replication in hepatocytes; of the cytokines, IL-1*β* is the most effective in inhibiting established HBV infection in vitro [147,148]. Additionally, TNF-*α* has been demonstrated to prevent the replication of various viruses, such as PRRSV and classical swine fever virus (CSFV) [149]. Various viruses employ varied mechanisms for TNF-*α* to limit viral infection; however, these mechanisms primarily involve the modulation of signaling pathways along with the inhibition or stimulation of receptor expression [150]. IL-6 plays a crucial role in maintaining homeostasis, being promptly produced upon disruption of homeostasis or tissue damage. It effectively aids the host in defending against emergent stress by triggering an acute-phase immune response [151]. The antiviral mechanism of other cytokines is rarely reported, which provides a direction for the next step of studying M*φ* polarization.

In contrast, M2 M*φ*s secrete anti-inflammatory cytokines, such as IL-10, to suppress both inflammatory and immune responses, thereby attenuating viral replication stimulation [152]. Normally, the biological function of M2 M*φ*s is to facilitate tissue healing while exhibiting an anti-inflammatory role [153].

### 5.3. Activation of Other Immune Cells

M1 M*φ*s recruit additional immune cells to the site of infection through the release of pro-inflammatory cytokines, thus forming a microenvironment conducive to viral replication and spread, which can cause tissue damage [154]. The M*φ*-derived cytokines IL-2 and IL-12 further promote T-cell activation, while M1 M*φ*s can also secrete IL-1*β*, IFN-*β*, or IL-15 to enhance the cytotoxicity of NK cells, which play key roles in destroying infected cells and inhibiting further viral replication. Activation of T cells by M1 M*φ*s can subsequently trigger the downstream antiviral response, which primarily relies on the elevated expression levels of CD80 and CD86 in M1 M*φ*s [76].

However, within tumor tissues, M2 M*φ*s can exert immunosuppressive effects on T-cell functionality by inhibiting anti-tumor immune responses and disrupting intercellular interactions among immune cells, thereby promoting tumor progression [155]. A study demonstrated a close association between CD155 molecule expression in colorectal cancer (CRC) and the transformation of tumor-associated M*φ*s into the M2 phenotype. The presence of the CD155 molecule elicits a phenotypic transition in M*φ*s, shifting them from a pro-inflammatory and anti-cancer state to an immunosuppressive and tumor-promoting state.

### 5.4. Cell Softness and Phagocytosis

For most viruses, M1 M*φ*s play an antiviral role through the aforementioned mechanisms, while M2 M*φ*s promote viral replication. For some viruses, such as SARS-CoV-2, additional mechanisms exist to regulate viral replication. M1 M*φ*s can phagocytose virus-infected cells or cellular debris and transfer viruses to other immune cells, thus promoting viral spread. According to a current investigation, SARS-CoV-2 transmission is facilitated by classically active M1 alveolar M*φ*s (AMs); on the other hand, diffusion is restricted by alternatively activated M2 AMs [156]. Researchers have observed that the heightened cellular softness of M1 M*φ*s renders them more susceptible to phagocytic virus entry into the cell. In certain respects, M2 M*φ*s can also exert antiviral effects. Firstly, they possess the capability to eliminate virus-infected cells or cellular debris through phagocytosis, thereby diminishing viral dissemination. Of note, mechanical softness serves as a metric for deformability, and research has indicated that tumorigenic cells exploit deformability to efficiently engulf particles [124]. Studies have demonstrated that M2 M*φ*s exhibit reduced cellular softness, which impedes the phagocytic uptake of invading viruses [156].

### 5.5. pH Homeostasis

Upon entering the cell, viruses undergo degradation within the lysosome and endosome systems. The increased acidity in the endosome facilitates disruption of the viral envelope by the SARS-CoV-2 S protein, leading to fusion with the cell membrane. Following fusion, the viral genome overcomes nucleosomal restriction and enters the cytoplasm to initiate the viral replication process [157]. The activation of low-pH-dependent cathepsin (CTSL) in the endosome causes the membrane of the viral particle and the endosome to split at the contact site by cutting the viral spike protein, resulting in the release of viral RNA into the cytoplasm [158]. Notably, the acidic environment generated by M1 M*φ*s may induce destabilization of viral particles, thereby facilitating the secretion of viral RNA into the cytoplasm for replication processes. The alkaline endosomal cyst cavity of M2 M*φ*s may inhibit the separation of viral genetic material RNA from its particle components, thereby retaining the virus within the endosome and preventing it from breaching the endosome’s barrier to enter the cytoplasm and initiate the replication process.

Subsequently, the late endosome fuses with the vesicles of the Golgi-derived hydrolase to form lysosomes, and then, M2 M*φ*s deliver the endosome capsule containing the virus to the acidic lysosomes for degradation. One study found that in cells infected with mouse hepatitis virus (MHV), protease activity in the lysosome was reduced by 40% [159]. There is evidence that for TAMs, their lysosomal degradative capacity relies on the lysosomal acidity of opposing phenotypes [160], wherein M1 M*φ*s have an optimal lysosomal activity that triggers the subsequent antigen cross-presentation for the activation of CD8^+^ T cells. In contrast, M2 TAMs possess more potent lysosomal acidity and protease activity, which readily gives rise to antigen degradation, restricts effective antigen cross-presentation, and results in immune silencing [161,162]. Therefore, the specific polarization of M2 TAMs toward the M1 phenotype holds significant potential to reverse the immunosuppressive tumor microenvironment, enhance antigen cross-presentation, and ultimately achieve robust cancer immunity [163]. In summary, both M1 and M2 M*φ*s can exert inhibitory effects on viral replication and transmission through various mechanisms, playing crucial roles in the antiviral immune response. However, excessive activation of M2 M*φ*s may also give rise to issues, such as immune response suppression or tissue damage. By employing mechanisms associated with the endosomal lysosome pathway, researchers have devised a novel targeting strategy that is capable of facilitating alveolar M*φ*s to engulf more viruses. The oxidized cholesterol carried by microparticles inhibits the internal small body sub-pump, rendering its cyst cavity more alkaline. This can prevent the separation of the SARS-CoV-2 RNA from the virion particle components and thereby allow it to be delivered to the lysosome for degradation [164].

## 6. Conclusions and Outlooks

This review mainly summarizes the mechanism underlying the polarization of different M*φ* phenotypes, highlighting that M*φ* polarization represents a promising area for further investigation. M*φ* polarization is a dynamic and reversible process that plays crucial roles in the pathogenesis, progression, and prognosis of numerous immune response-related disorders. Currently, only a limited number of studies have reported the antiviral effects associated with M*φ* plasticity and lysosomal pH modulation during viral infection; however, this aspect deserves further investigation as a potential target of interest.

NK cells are an important component of innate immunity, playing a key role in host defense through their ability to release cytokines and mediate the cytolytic activity against tumor cells and virus-infected cells, thereby enabling them to recognize and directly eliminate various distressed cell types [165]. Dendritic cells (DCs) are distinctive hematopoietic cells that interconnect innate and adaptive immune responses, serving as the most efficacious antigen-presenting cells for the identification of pathogens [166]. Despite NK cells and DCs being highly efficient in eliminating pathogens, they still have limitations. The lifespan of NK cells is relatively short, which limits their sustained killing ability in the body. Additionally, the ability of NK cells to proliferate in the body is limited, which affects their effectiveness as a long-term immune response. Meanwhile, DCs can induce tumor immune tolerance, which may limit their effectiveness in anti-tumor immunotherapy. In comparison with other immune cells, M*φ*s possess a distinct function; they serve as a primary target of numerous pathogens while demonstrating unique advantages in viral clearance. M*φ*s demonstrate high plasticity, which allows them to adapt effectively to diverse immune requirements [167]. M*φ*s, as professional phagocytes, play an important role in clearing infectious agents by internalizing and degrading pathogens, as well as engulfing apoptotic cells. They mediate phagocytosis through surface phagocytic receptors, including mannose receptors and scavenger receptors. During the early stages of viral infection, M*φ*s are polarized into M1 M*φ*s to combat pathogens, and later, M2 M*φ*s are increased to promote tissue repair. This dynamic polarization is crucial for the entire antiviral process and subsequent recovery.

Nowadays, the escalating prevalence of diverse respiratory viral infections has prompted our attention toward elucidating the pivotal role of M*φ*s in orchestrating host responses against respiratory viruses. Now that coronavirus disease 2019 (COVID-19) poses a serious threat to global public health [168], we have learned that different phenotypes of M*φ* polarization exhibit specific effects on SARS-CoV-2. M1 M*φ*s can promote virus spread while M2 M*φ*s may limit it. AMs are among the first targets of SARS-CoV-2 infection; however, their precise response to virus attack is still unclear. AMs constitute a distinct subgroup of lung M*φ*s and serve as the primary defense against foreign invasion in lung tissue. These M*φ*s are crucial components of the airway and mucosa, playing a pivotal role in disease pathogenesis [169]. M1-like M*φ*s are potent producers of antiviral interferons and more resistant to Rhinovirus (RV) infection [170].

African swine fever (ASF), caused by ASFV, is a severe and detrimental disease posing a significant threat to the global pig industry [171]. Similar to SARS-CoV-2, ASFV also targets M*φ*s; therefore, M*φ*s can provide us with an understanding of two of the biggest challenges in the world. Infection with ASFV suppresses the host immune response, but the underlying mechanism remains poorly understood due to existing limitations in our knowledge regarding both ASFV and macrophage biology. It is known that M*φ*s are an important target of ASFV’s attack. Therefore, investigations into the functions and mechanisms of M*φ* polarization will provide important insights for the prevention and control of ASF.

M*φ*s, a population of immune cells with strong heterogeneity and plasticity, play dually regulatory roles in the progression and regression of inflammatory responses induced by viral infections. Thus, M*φ*s represent a promising target for the treatment of viral diseases. Virus-infected M*φ*s are usually polarized into a pro-inflammatory M1 phenotype during early stages of infection while transitioning into an anti-inflammatory M2 phenotype later on. Early-stage M1 M*φ*s can exert powerful antiviral functions, while late-stage M2 M*φ*s contribute positively to tissue repair and regeneration. Exploring potential mechanisms aimed at inhibiting early-stage M2 polarization as well as late-stage M1 polarization could provide deeper insights into the interplay between viruses and macrophages. Elucidating the roles of M*φ* polarization throughout viral infections along with their associated regulatory mechanisms will offer novel perspectives on pathogenesis and treatment strategies for viral infectious diseases.

## Figures and Tables

**Table 1:** Function of macrophages in different tissues.

Cell Types	Distribution	Functions	References
Microglial cells	Central nervous system	Engulf synaptic material; produce growth factors	[15]
Alveolar macrophages	Lung	Phagocytose bacteria and particulates; scavenge and degrade lung surfactant; preserve airway integrity; involved in pro-inflammatory response and immunosuppression	[16]
Kupffer cells	Liver	Take up circulating senescent or damaged red blood cells; involved in iron metabolism; recycle iron from hemoglobin via ferroportin transporter	[17]
Red-pulp macrophages	Spleen	Phagocytose damaged and aged red blood cells and blood-derived particles; maintain blood homeostasis	[18]
Marginal-zone macrophages		Participate in immune responses	[19]
Intestine macrophages	Intestine	Phagocytic pathogen; capture dietary material; regulate smooth muscle contractions, control peristalsis; regulate gut motility	[20,21]
Peritoneal macrophages	Peritoneum	Protecting the peritoneal cavity against microorganisms and inflammation; support functions of B cells	[22]
Langerhans cells	Skin	Populate epidermis; first line of defense against exogenous pathogen invasion	[23]
Osteoclast	Bone	Dissolve bone mineral and enable continuous remodeling of bone matrix; stimulate osteoblast activity and bone formation	[24]

**Table 2:** The phenotypes and mechanisms of macrophage polarization.

Cell Types	Polarization Stimuli	Surface Markers	Cytokines	Signaling Cascades	References
M1	IFN-*γ*, LPS, and GM-CSF	CD80, CD68, CD86, CD32, CD64, iNOS, MHC-II, IL-1R, TLR-2, and TLR-4	iNOS, TNF-*α*, IL-1*β*, IL-12, IL-18, IL-23, IL-6, and IL-1*α*	JAK/STAT1, NF-*κ*B, and Notch	[31,32,33,34,35,36,37,38,39,40,41,42]
M2a	IL-4 and IL-13	CD206, CD68, CD163, and Arg-1	Arg-1, IL-10, TGF-*β*, and IL-1*β*	JAK/STAT6	[29,43,44,45,46,47,48,49]
M2b	TLR ligands and IL-1*β*	CD86, CD68, and CD206	IL-10, IL-1*β,* IL-6, and TNF-*α*	PI3K/AKT	[45,46,47,48,50]
M2c	IL-10 and TGF-*β*	CD163, CD68, CD206, and Arg-1	IL-10, Arg-1, and TGF-*β*	JAK/STAT3, and NF-*κ*B	[45,46,47,48,51]
M2d	TLR antagonists	CD68 and CD206	IL-10	NF-*κ*B	[45,46,47,48,52]
TAMs	IFN-*γ*, IL-4, and IL-13	CD163, CD206, and CD81	iNOS, IL-10, TGF-*β*, CCL2, and CCL5	JAK/STAT1, NF-*κ*B, and PI3K	[53,54]
CD169^+^	G-CSF	CD169, CD11b, MHC-II, CD68, CD206, and VCAM-1	IL-10 and CCL22	/	[55]
TCR^+^	Tumor microenvironment	TCR-*α*, TCR-*β*, TCR-*γ*, TCR-*δ*, and CD3	CCL2	/	[56]
M4	CXCL4	CD163 and CD206	TNF-*α* and CCL18	/	[57]
Mox	QxPAPC	HO-1, Srxn1, Gclc, and Gclm	IL-1*β* and VEGF	Nrf2 and TLR2	[58]
M(Hb)	Hemoglobin	CD163 and CD206	IL-10 and IL-1R	PI3K/AKT	[59]
